# Laparoscopic conversion in colorectal cancer surgery; is there any improvement over time at a population level?

**DOI:** 10.1007/s00464-018-6042-2

**Published:** 2018-01-17

**Authors:** Michael P. M. de Neree tot Babberich, Julia T. van Groningen, Evelien Dekker, Theo Wiggers, Michel W. J. M. Wouters, Willem A. Bemelman, Pieter J. Tanis, On Behalf of the Dutch Surgical Colorectal Audit

**Affiliations:** 1 Department of Surgery, Academic Medical Center, Amsterdam, The Netherlands; 2 Department of Gastroenterology and Hepatology, Academic Medical Center, Meibergdreef 9, 1105 AZ Amsterdam, The Netherlands; 3 Department of Surgery, Leiden University Medical Center, Leiden, The Netherlands; 4 Department of Surgery, University Medical Center Groningen, Groningen, The Netherlands; 5 Department of Surgical Oncology, Netherlands Cancer Institute-Antoni van Leeuwenhoek, Amsterdam, The Netherlands; 6 Scientific Bureau, Dutch Institute for Clinical Auditing, Leiden, The Netherlands

**Keywords:** Colorectal cancer, Laparoscopic surgery, Conversion, Improvements, Learning

## Abstract

Conversion of laparoscopic colorectal cancer resection has been associated with worse outcome, but this might have been related to a learning curve effect. This study aimed to evaluate incidence, predictive factors and outcomes of laparoscopic conversion after the implementation phase of laparoscopic surgery at a population level. Patients undergoing elective resection of non-locally advanced, non-metastatic colorectal cancer between 2011 and 2015 were included. Data were extracted from the Dutch Surgical Colorectal Audit. Patients were grouped as laparoscopic completed (LR), laparoscopic converted (CONV) with further specification of timing (within or after 30 min) as registered in the DSCA, and open resection (OR). Uni- and multi-variate analyses were used to determine predictors of conversion and outcome (complicated course and mortality), with evaluation of trends over time. A total of 23,044 patients with colon cancer and 11,324 with rectal cancer were included. Between 2011 and 2015, use of laparoscopy increased from 55 to 84% in colon cancer, and from 49 to 89% in rectal cancer. Conversion rates decreased from 11.8 to 8.6% and from 13 to 8.0%, respectively. Laparoscopic hospital volume was independently associated with conversion rate. Only for colon cancer, the rate of complicated course was significantly higher after CONV compared to OR (adjusted odds ratio 1.486; 95% CI 1.298–1.702), and significantly higher after late (> 30 min) compared to early conversion (adjusted odds ratio 1.341; 1.046–1.719). There was no impact of CONV on mortality in both colon and rectal cancer. The use of laparoscopic colorectal cancer surgery increased to more than 80% at a national level, accompanied by a decrease in conversion which is significantly related to the laparoscopic hospital volume. Conversion was only associated with complicated course in colon cancer, especially when the reason for conversion consisted of an intra-operative complication, without affecting mortality.

**Electronic supplementary material:**

The online version of this article (10.1007/s00464-018-6042-2) contains supplementary material, which is available to authorized users.

Laparoscopic surgery is increasingly used as standard of care for colorectal cancer resection. There is still wide variability among countries in the use of laparoscopy and a still existing controversy about oncological safety in rectal cancer resections [1–6]. Besides the short-term advantage of laparoscopic surgery with faster postoperative recovery, there is increasing evidence showing a lower risk of small bowel obstruction and incisional hernia in the long run [7, 8].

With the increasing use of laparoscopy for colorectal cancer, there has been a growing concern about the possible negative outcomes of conversion to open surgery, especially reactive conversion [9, 10]. Contradictory findings have been reported on the influence of conversion on morbidity and oncological outcome. This is partly explained by the comparisons with either the successfully completed laparoscopy or the open resection group, besides differences in casemix and surgical experience. Reported conversion rates vary largely in RCTs and can be as high as 29% [11–15]. Several patient- and tumor-related risk factors for conversion have been identified, such as BMI, ASA, left-sided and sigmoid tumors, pT4 stage, acute surgery, metastatic setting, sex, age, and hospital volume [16–18].

Laparoscopy for colorectal cancer resection has been introduced in the Netherlands with structured training programs, which resulted in fast and successful implementation [2, 19]. The detailed perioperative data from all Dutch centers performing colorectal cancer surgery, as registered in the Dutch Surgical Colorectal Audit (DSCA), enable a detailed analysis of laparoscopic conversion during the last phase of the implementation process.

Therefore, the purpose of this population-based analysis was to study conversion of laparoscopy to open surgery for colorectal cancer over time, determining risk factors for conversion and predictors of short-term postoperative outcome.

## Methods

Data were derived from the DSCA, a disease-specific national audit [20]. This audit collects information on patient, tumor, treatment, and short-term outcome characteristics of all patients undergoing a resection for primary colorectal cancer in the Netherlands.

### Patients

For this study, no ethical approval or informed consent was required under Dutch law. The status of laparoscopic colorectal cancer surgery in the Netherlands in 2010 has been previously published [2]. All patients (*n* = 51,511) who underwent resection since then (between January 1st, 2011 and December 31th, 2015) were considered potentially eligible. Minimal data requirements were information on tumor location, date of surgery and 30-day/in-hospital mortality, which was available for 51,284 patients. Also, patients with locally advanced or metastatic tumors were excluded because of the high risk of allocation bias. Laparoscopic surgery in locally advanced tumors with their corresponding conversion rates for colon cancer was published previously [21]. Furthermore, for the purpose of this study, only patients that underwent surgery in the elective setting were selected.

### Data extraction and outcome parameters

The following data were extracted from the DSCA database: patient and disease characteristics, procedural characteristics and postoperative outcome within 30 days after resection or in-hospital events. Conversion is further specified in the DSCA into early (≤ 30 min) and late (> 30 min) conversion. This arbitrary cutoff was chosen at the initiation of the DSCA, because of limited relevant literature at that time [9]. This is not according to the recently achieved international consensus about sub classifying conversion into strategic and reactive.[22] However, reasons for early or late conversion are registered, enabling to identify reactive conversions.

Previous abdominal surgery, being a risk factor for conversion, is available but not further specified in the DSCA and includes for example laparoscopic appendectomy and prior open bowel resection.

Outcome parameters were postoperative mortality (< 30 days or in-hospital) and complicated postoperative course, defined as a postoperative complication resulting in a hospital stay > 14 days and/or a reintervention and/or mortality. Hospitals were categorized into low-volume (< 30), medium volume (30–50) and high volume (> 50) based on the average number of laparoscopic colon cancer resections per hospital per year, and low-volume (< 20), medium volume (20–30) and high volume (> 30) based on the average number of laparoscopic rectal cancer resections per hospital per year. Hospitals were further categorized into non-teaching, teaching and academic.

### Data analysis

Analyses were performed separately for colon and rectal cancer. To evaluate trends over time, data were reported for each year of registration in the DSCA and tested for statistical significance. Open resection (OR), laparoscopic completed resection (LR), and laparoscopic converted resection (CONV) were classified based on how the procedure started (open or laparoscopic). To analyze a hospital volume–conversion relationship, data were aggregated on hospital level for each of all 92 hospitals in the Netherlands over the years 2011–2015. This was done on an intention-to-treat basis by including conversions in the laparoscopic group. Group comparisons were performed using multivariable logistic regression analysis for dichotomous variables and Mann–Whitney *U* test for continuous variables.

Risk factors for conversion, including laparoscopic hospital volume and types of surgical procedures, were determined using univariable and multivariable analyses. A casemix adjusted scatterplot was made to show laparoscopic hospital volume in relation to corresponding conversion rate. The impact of conversion, timing of conversion, and reason of conversion on outcome parameters was evaluated using both univariable and multivariable logistic regression analyses.

The following factors were included in the multivariable analysis to adjust for differences in casemix; gender, age, American Society of Anesthesiologists (ASA) score, Charlson comorbidity score, Body Mass Index (BMI), any pre-operative complication, pT-classification. For colon cancer, the location of the tumor within the colon was added to the casemix and for rectal cancer the casemix was expanded with tumor distance from anal verge, cT-classification, pre-operative radiotherapy (no radiotherapy, short course radiotherapy or chemoradiotherapy), and surgical procedure (Low Anterior Resection, Abdominal Perineal Resection or different). Further details on casemix correction are described in previous studies [23]. Because we were interested in conversion and the outcomes of conversion over the years, we also included the risk factor previous abdominal surgery and the year of operation to the standard casemix. If conversion was the outcome of interest, hospital volume and type of hospital was also added to the multivariable model.

A *p* value of less than 0.05 was considered statistically significant. All analyses were performed in SPSS 24.0 Statistics for Windows (Armonk, NY: IBM Corp).

## Results

### Baseline characteristics

**Fig. 1:**
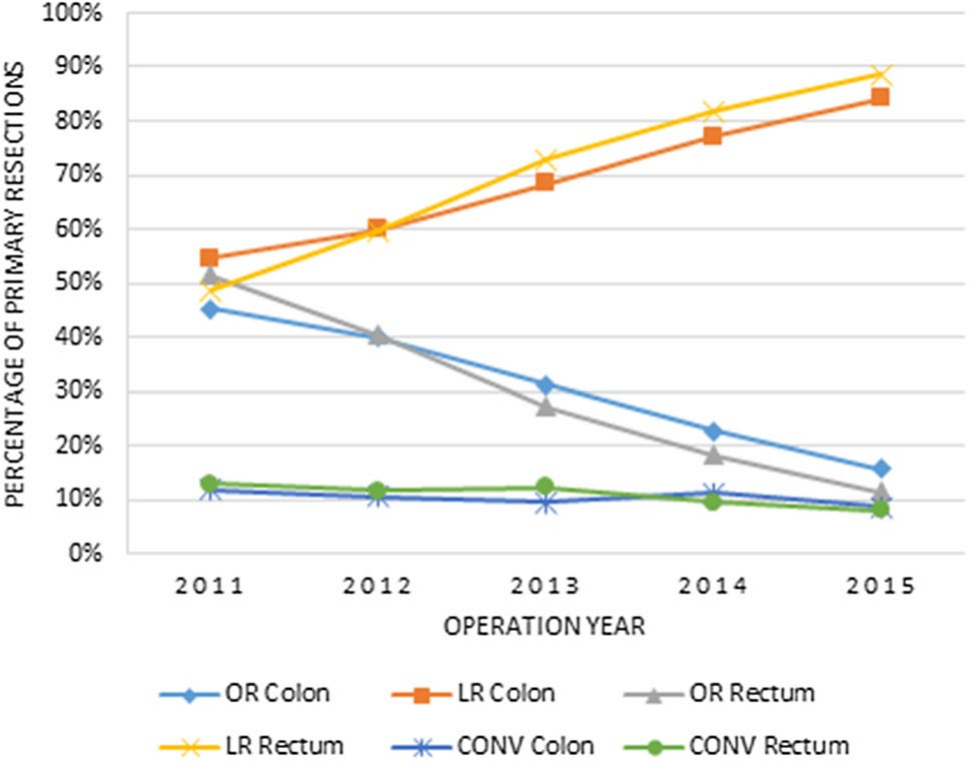
Percentage of open resections (OR), laparoscopic resections (LR), and laparoscopic converted (CONV) resections for primary colorectal carcinoma over the years 2011–2015, separated for colon and rectum

A total of 34,368 patients were included for analysis, of whom 23,044 (67.1%) underwent resection for colon cancer and 11,324 (32.9%) for rectal cancer. Figure 1 shows the percentages of laparoscopic (including conversions) and open surgery for colon and rectal cancer in the Netherlands between 2011 and 2015. An absolute increase in laparoscopic surgery for colon cancer of 29% and for rectal cancer of 40% was seen since 2011. Patient characteristics of the OR, LR and CONV groups are displayed separately for colon and rectum in Table 1.

**Table 1:** Patient-, tumor-, and surgery-baseline characteristics

		Colon					Rectum				
			Laparoscopic resections					Laparoscopic resections			
		OR	LR	CONV	p*	p*	OR	LR	CONV	p*	p*
	Total *N*	6800	14601	1643	CONV vs. LR	CONV vs. OR	3250	7231	843	CONV vs. LR	CONV vs. OR
		*n* (%)	*n* (%)	*n* (%)			*n* (%)	*n* (%)	*n* (%)		
Patient											
Age	75 +	3143 (46)	5131 (35)	649 (40)	< 0.001	< 0.001	903 (28)	1901 (26)	265 (31)	< 0.001	0.037
Gender	Male	3548 (52)	7876 (54)	1044 (64)	< 0.001	< 0.001	2161 (67)	4549 (63)	619 (73)	< 0.001	< 0.001
ASA Score	I–II	4803 (71)	11,677 (80)	1167 (71)	< 0.001	n.s.	2605 (80)	6215 (86)	668 (79)	< 0.001	n.s.
	III	1869 (28)	2790 (19)	456 (28)			616 (19%)	984 (14)	168 (20)		
	IV–V	110 (1.6)	125 (0.9)	19 (1.2)			25 (0.8)	32 (0.4)	7 (0.8)		
Charlson Score	0	2882 (42)	7530 (52)	674 (41)	< 0.001	n.s.	1679 (52)	4300 (60)	445 (53)	< 0.001	n.s.
	1	1592 (23)	3413 (23)	435 (27)			711 (22)	1492 (21)	186 (22)		
	2	1230 (18)	2114 (15)	282 (17)			491 (15)	913 (13)	129 (15)		
	3 +	1096 (16)	1544 (11)	252 (15)			369 (11)	526 (7.3)	83 (9.8)		
BMI (kg/m^2^)	< 18.5	121 (1.8)	153 (1)	14 (0.9)	< 0.001	< 0.001	52 (1.6)	81 (1.1)	5 (0.6)	< 0.001	< 0.001
	18.5–25	2545 (37)	5472 (37)	440 (27)			1151 (35)	3005 (42)	196 (23)		
	25–30	2622 (39)	6076 (42)	660 (40)			1346 (41)	2991 (41)	389 (46)		
	30 +	1216 (18)	2568 (18)	488 (30)			570 (18)	1011 (14)	241 (29)		
	Unknown	296 (4.3)	332 (2.3)	41 (2.4)			131 (4)	143 (2)	12 (1.4)		
Previous abdominal surgery	Yes	2890 (43)	4656 (32)	696 (43)	< 0.001	n.s.	1121 (35)	1904 (26)	288 (34)	< 0.001	n.s.
Tumor											
Location of tumor	Ascending colon up to and including hepatic flexure	3674 (54)	5835 (40)	607 (37)	< 0.001	< 0.001					
	Transverse colon up to and including splenic flexure	886 (13)	1063 (7.2)	198 (12)							
	Descending colon	432 (6.4)	801 (5.4)	140 (8.5)							
	Sigmoid colon	1808 (27)	6902 (47.4)	698 (43)							
Distance from anal verge	≤ 5 cm						1297 (42)	2350 (34)	220 (27)	< 0.001	< 0.001
	6–10 cm						1228 (40)	2845 (41)	407 (50)		
	> 10 cm						570 (18)	1789 (26)	187 (23)		
Pre-operative tumor complications	Yes	2145 (32)	3923 (27.1)	507 (31)	< 0.001	n.s.	835 (26)	1413 (20)	219 (26)	< 0.001	n.s.
Pathological T stage	T1	546 (8)	2037 (14)	178 (11)	< 0.001	< 0.001	248 (7.6)	859 (12)	60 (7.1)	< 0.001	0.023
	T2	1301 (19)	3504 (24.2)	327 (20)			1138 (35)	2481 (34)	265 (32)		
	T3	4816 (71)	8810 (60.1)	1117 (68)			1539 (47)	3274 (45)	451 (54)		
	T0	16 (0.4)	85 (0.6)	6 (0.4)			258 (8)	537 (7.4)	56 (6.7)		
	Unknown	115 (1.7)	165 (1.1)	15 (0.8)			18 (1.9)	78 (1.1)	11 (1.3)		
Surgery											
Procedure	Ileocecal resection	74 (1.1)	88 (0.6)	9 (0.6)	< 0.001	< 0.001					
	Right hemicolectomy	3765 (56)	5962 (41)	634 (39)							
	Transverse colectomy	287 (4.2)	200 (1.4)	29 (1.8)							
	Left hemicolectomy	767 (11)	1337 (9.1)	265 (16)							
	Sigmoid resection or (Low) anterior resection	1709 (25)	6775 (46.7)	658 (40)			2165 (67)	5323 (74)	683 (81)	< 0.001	< 0.001
	Abdominoperineal resection						1015 (31)	1822 (25)	147 (18)		
	Not further specified	163 (2.4)	186 (1.3)	39 (2.4)			49 (1.7)	60 (0.8)	10 (1.2)		
Anastomosis and stoma	Anastomosis without stoma	6056 (91)	13,589 (94.2)	1438 (89)	< 0.001	< 0.001	447 (14)	1730 (24)	127 (16)	< 0.001	< 0.001
	Anastomosis with stoma	196 (2.9)	350 (2.4)	79 (4.9)			1070 (34)	2617 (37%)	360 (44)		
	Stoma without anastomosis	425 (6.4)	475 (3.4)	104 (6.4)			1613 (52)	2734 (39)	334 (41)		

*OR* open resection, *LR* laparoscopically completed resection, *CONV* laparoscopic converted to open

*Chi-square test was used for all categorical variables. *n.s*. not significant (*p* > 0.05)

### Incidence and risk factors of conversion

During the study period, conversion rates significantly decreased from 11.8 to 8.6% for colon cancer, and from 13 to 8.0% for rectal cancer (Table 2). The proportions of early and late conversions did not change significantly.

**Table 2:** Time trends (2011–2015) for OR and LR of postoperative complicated course, mortality and for LR also conversion rate

	Year of surgery					*p* for trend
	2011	2012	2013	2014	2015	
*Colon*						
Open resection, no. of patients	1723	1708	1297	1163	909	< 0.001
Complicated course	335 (19.4%)	301 (17.6%)	232 (17.9%)	205 (17.6%)	134 (14.7%)	0.009
Mortality	66 (3.8%)	69 (4%)	43 (3.3%)	38 (3.3%)	16 (1.8%)	0.006
Laparoscopic resection, no. of patients	2081	2569	2836	3931	4827	< 0.001
Complicated course	304 (14.6%)	326 (12.7%)	352 (12.4%)	467 (11.9%)	527 (10.9%)	< 0.001
Mortality	63 (3%)	48 (1.9%)	38 (1.3%)	52 (1.3%)	53 (1.1%)	< 0.001
Conversion, no. of patients^a^	245 (11.8%)	273 (10.6%)	271 (9.6%)	441 (11.2%)	413 (8.6%)	< 0.001
Early conversion (≤ 30 min)	7.1%	8.0%	7.0%	7.5%	6%	
Late conversion (> 30 min)	4.7%	2.6%	2.6%	3.8%	2.6%	
% Early within conversion	60%	75%	73%	66%	70%	0.457
*Rectum*						
Open resection, no. of patients	1053	901	574	429	293	< 0.001
Complicated course	280 (26.6%)	244 (27.1%)	146 (25.4%)	94 (21.9%)	63 (21.5%)	0.017
Mortality	39 (3.7%)	15 (1.7%)	13 (2.3%)	8 (1.9%)	5 (1.7%)	0.025
Laparoscopic resection, no. of patients	994	1334	1546	1929	2271	< 0.001
Complicated course	211 (21.2%)	261 (19.6%)	285 (18.4%)	380 (19.7%)	431 (19%)	0.28
Mortality	27 (2.7%)	21 (1.6%)	14 (0.9%)	19 (1%)	25 (1.1%)	0.001
Conversion, no. of patients^a^	129 (13.0%)	156 (11.7%)	191 (12.4%)	185 (9.6%)	182 (8.0%)	< 0.001
Early conversion (≤ 30 min)	5.5%	6.7%	6.7%	5%	4%	
Late conversion (> 30 min)	7.4%	5%	5.6%	4.6%	4.1%	
% Early within conversion	43%	57%	54%	52%	49%	0.673

^a^For laparoscopic surgery also the conversion rates are shown, separated in early and late conversion

Using univariable analysis, the CONV groups for both colon and rectum appeared to have a higher age (75 +), were more often male, ASA III+, had more often a Charlson comorbidity score 3 +, BMI 30 + and more often previous abdominal surgery, compared to LR (Table 1). The risk for conversion was further analyzed regarding surgical procedure and laparoscopic hospital volume.

**Fig. 2:**
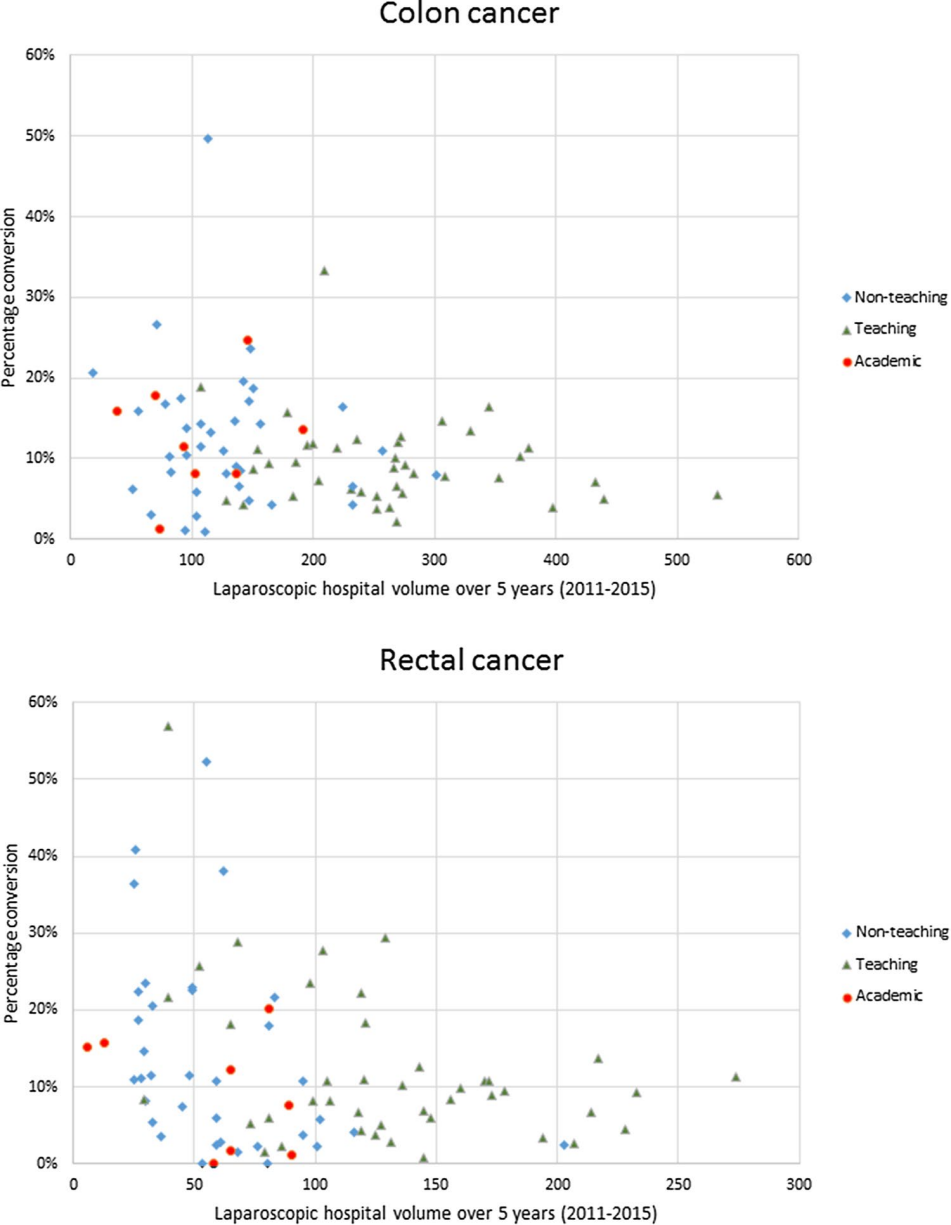
Total laparoscopic hospital volume from 2011 to 2015 plotted against corresponding conversion rate for colon and rectal cancer, adjusted for casemix

The surgical procedure of the colon with the highest risk of conversion was the left hemicolectomy with an adjusted odds ratio of 1.960 [95% confidence interval (CI) 1.670–2.300]. For rectal cancer, the location with the highest risk for conversion was 6–10 cm distance from the anal verge (adjusted odds ratio 1.329, CI 1.095–1.613) (Table S1). Figure 2 shows the total laparoscopic hospital volume plotted against corresponding conversion rate for colon and rectal cancer. Four hospitals were excluded from analysis because no LR was performed or LR was stopped in 2015 or before. After adjusting for casemix, the risk of conversion in colon cancer was lower in the high laparoscopic hospital volume (adjusted odds ratio 0.718, CI 0.605–0.852) compared to the low-volume group. In rectal cancer, the risk of conversion was lower in medium and high laparoscopic hospital volumes (adjusted odds ratio 0.573, CI 0.465–0.707 and adjusted odds ratio 0.419, CI 0.338–0.520, respectively), compared to the low-volume group (Table S2). Multivariable subanalysis for different types of hospital showed a higher casemix adjusted odds on a laparoscopic approach in a teaching hospital (adjusted odds ratio 1.224, CI 1.147–1.307) compared to non-teaching hospitals for colon cancer. This was not significant for the academic hospitals compared to the non-teaching hospitals. If a laparoscopic approach was chosen, the casemix adjusted odds on conversion were not different between types of hospitals for colon cancer. For rectal cancer, the odds on a laparoscopic approach were higher in teaching hospitals (adjusted odds ratio 1.449, CI 1.313–1.599) and lower in academic hospitals (adjusted odds ratio 0.661, CI 0.553–0.791) compared to non-teaching hospitals. The odds on laparoscopic conversions were found to be slightly higher in teaching hospitals (adjusted odds ratio 1.307, CI 1.076–1.589) and lower in academic hospitals (adjusted odds ratio 0.552, CI 0.374–0.914). Reasons for conversion in both colon and rectal cancer were not significantly different (*p* = 0.054) between different types of hospitals.

Table 3 shows that the leading cause for conversion was exposure difficulties, both in the early and the late conversion group, with significantly more often intra-operative complication as reason for late conversion compared to early conversion (colon *p* < 0.001, rectum *p* = 0.003). The most common intra-operative complications consisted of; ‘bleeding for which transfusion was required’ (19.9%), ‘intestinal trauma for which a reintervention was required’ (12.9%) and ‘other, not further specified’ (38.2%).

**Table 3:** Time trends (2011–2015) of reasons for early and late conversion for colon and rectum

	Timing conversion	Reason of conversion	Year of surgery				
			2011	2012	2013	2014	2015
Colon	Early (≤ 30 min) conversion	Extensiveness (%)	13.8	10.3	13.2	8.9	14.1
		Exposure difficulties (%)	82.1	83.7	83.8	89.7	81.2
		Intra-operative complication (%)	4.1	5.9	3	1.4	4.7
	Late (> 30 min) conversion	Extensiveness (%)	13	9.8	13.6	8.6	9.6
		Exposure difficulties (%)	72.8	68.9	66.7	69	72.8
		Intra-operative complication (%)	14.1	21.3	19.7	22.4	17.5
Rectum	Early (≤ 30 min) conversion	Extensiveness (%)	3.6	11.2	12.7	3.2	7.1
		Exposure difficulties (%)	90.9	85.4	85.3	89.5	87.1
		Intra-operative complication (%)	5.5	3.4	2	7.4	5.9
	Late (> 30 min) conversion	Extensiveness (%)	12.7	13.6	10.8	6.7	10.3
		Exposure difficulties (%)	77.5	78.8	79.5	81.3	77
		Intra-operative complication (%)	9.9	7.6	9.6	12	12.6

### Outcomes

The overall percentages of complicated course and mortality per registration year are displayed in Table 2, showing a decrease of both outcome parameters for laparoscopic (including conversion) as well as open resection.

In colon cancer, CONV compared to OR had a slightly higher risk of anastomotic leakage or abscess at the level of the anastomosis (Table 4). Also, conversion in colon cancer was associated with a higher crude percentage of intra- and post-operative (surgical) complications, a higher crude complicated course, and a longer hospital stay. In rectal cancer, conversion was significantly associated with more intra-operative complications, higher crude proportion of complicated course, and longer hospital stay in comparison with the OR group (Table 4).

**Table 4:** Intra- and post-operative outcomes of OR, LR, and CONV

		Colon					Rectum				
			Laparoscopic resections					Laparoscopic resections			
		OR	LR	CONV	p*	p*	OR	LR	CONV	p*	p*
	Total *N*	6800	14601	1643	CONV vs. LR	CONV vs. OR	3250	7231	843	CONV vs. LR	CONV vs. OR
		*n* (%)	*n* (%)	*n* (%)			*n* (%)	*n* (%)	*n* (%)		
Intra-operative complications and postoperative outcomes											
Intra-operative complication	No	6505 (97)	14,225 (99)	1447 (89)	< 0.001	< 0.001	3029 (95)	6995 (97.5)	724 (87)	< 0.001	< 0.001
	Blood loss with transfusion	30 (0.4)	34 (0.2)	42 (2.6)			37 (1.2)	22 (0.3)	18 (2.2)		
	Splenic trauma with splenectomy	16 (0.2)	9 (0.1)	3 (0.2)			3 (0.1)	3 (0)	2 (0.2)		
	Pancreatic/liver/choledochus/gallbladder trauma	8 (0.1)	5 (0)	7 (0.4)			3 (0.1)	2 (0)	1 (0.1)		
	Intestinal trauma for which surgical intervention	57 (0.9)	69 (0.5)	37 (2.3)			31 (1)	38 (0.5)	23 (2.8)		
	Ureter or Urethra trauma	12 (0.2)	10 (0.1)	10 (0.6)			14 (0.4)	33 (0.5)	11 (1.3)		
	Bladder trauma	6 (0.1)	20 (0.1)	1 (0.1)			5 (0.2)	11 (0.2)	5 (0.6)		
	Vaginal Trauma	0 (0)	0 (0)	2 (0.1)			10 (0.3)	11 (0.2)	1 (0.1)		
	Different	46 (0.7)	60 (0.4)	77 (4.7)			55 (1.8)	58 (0.8)	47 (5.7)		
Lymph nodes removed	10 +	6090 (90)	13,190 (90)	1457 (89)	n.s.	n.s.	2486 (77)	5826 (81)	688 (82)	n.s.	n.s.
Radical resection	R0	6650 (99)	14,378 (99.6)	1608 (99)	n.s.	n.s.	3109 (97)	7009 (97.8)	814 (97)	n.s.	n.s.
	R1	42 (0.6)	54 (0.4)	14 (0.9)			102 (3.2)	155 (2.2)	25 (3)		
	R2	15 (0.2)	8 (0,1)	3 (0.2)			7 (0.2)	4 (0.1)	0 (0)		
Circumferential margin	≤ 1 mm						162 (5)	268 (3.7)	25 (3)	n.s.	0.012
Complication within 30 days after operation	Yes	2167 (32)	3062 (21)	639 (39)	< 0.001	< 0.001	1365 (42)	2273 (32)	382 (45)	< 0.001	n.s.
If yes, general complication	Yes	1401 (65)	1817 (60)	405 (63)	n.s.	n.s.	829 (61)	1265 (56)	226 (59)	n.s.	n.s.
If yes, Surgical complication	Yes	1234 (58)	1859 (61)	411 (65)	n.s.	0.002	820 (62)	1460 (65)	249 (66)	n.s.	n.s.
Anastomotic leakage or abscess at anastomosis	Yes	359 (5.7)	665 (4.8)	111 (7.3)	< 0.001	0.021	141 (9.3)	374 (8.6)	35 (7.2)	n.s.	n.s.
Crude complicated course^a^	Yes	1207 (18)	1589 (11)	387 (24)	< 0.001	< 0.001	827 (25)	1324 (18)	244 (28.9)	< 0.001	0.039
If a reintervention was performed, what was the nature of reintervention	Radiologic	41 (6)	63 (5.5)	12 (5.2)	n.s.	n.s.	53 (12)	71 (8.1)	14 (10)	< 0.001	n.s.
	laparoscopic	8 (1.2)	219 (20)	2 (0.9)			10 (2.3)	212 (24)	6 (4.3)		
	Open	589 (86)	751 (70)	201 (87)			277 (64)	373 (43)	90 (64)		
	Different	47 (6.9)	57 (5.3)	17 (7.3)			96 (22)	222 (25)	30 (21)		
Crude mortality		232 (3.4)	210 (1.4)	44 (2.7)	n.s.	n.s.	80 (2.5)	87 (1.2)	19 (2.3)	0.011	n.s.
Median LOS (days) until discharge or death		10.1	7.5	11	< 0.001**	< 0.001**	12.5	9.7	13.9	< 0.001**	0.003**

*OR* open resection, *LR* laparoscopically completed resection, *CONV* laparoscopic converted to open

*Chi-square test was used for all categorical variables

**Mann–Whitney *U* test. *LOS* length of stay. *n.s*. not significant (*p* > 0.05)

^a^Postoperative complication resulting in a hospital stay > 14 days and/or a reintervention and/or mortality

For colon cancer, multivariable analysis revealed that conversion was independently associated with a significant higher risk of complicated course compared to the OR group [adjusted odds ratio 1.486 (CI 1.298–1.702)] (Table 5). By analyzing early and late conversion separately, an adjusted odds ratio of 1.352 (CI 1.153–1.586) and 1.814 (CI 1.465–2.245) was found, respectively. The risk of complicated course after late conversion was found to be significantly higher than after early conversion (adjusted odds ratio 1.341, CI 1.046–1.719). The risk of mortality did not differ significantly between CONV and OR.

**Table 5:** Uni- and multi-variate analysis for the association of OR, LR, and CONV with different timings of CONV on complicated course and mortality

	Odds ratio (CI)	Odds ratio (CI)	Odds ratio (CI)
	Univariate	Multivariate	Multivariate**
*Colon*^^^*			
Postoperative complicated course			
OR	Ref	Ref	Ref
LR	**0.566 (0.522–0.614)**	**0.668 (0.613–0.727)**	**0.706 (0.646–0.770)**
CONV	**1.428 (1.254–1.626)**	**1.423 (1.244–1.628)**	**1.486 (1.298–1.702)**
Early conversion	**1.319 (1.131–1.537)**	**1.292 (1.102–1.513)**	**1.352 (1.153–1.586)**
Late conversion	**1.685 (1.371–2.070)**	**1.746 (1.412–2.160)**	**1.814 (1.465–2.245)**
Late vs. early	**1.278 (1.004–1.626)**	**1.352 (1.055–1.732)**	**1.341 (1.046–1.719)**
Mortality (in-hospital or < 30 days)			
OR	Ref	Ref	Ref
LR	**0.413 (0.342–0.499)**	**0.589 (0.483–0.719)**	**0.680 (0.555–0.835)**
CONV	0.779 (0.562–1.080)	0.853 (0.609–1.195)	0.954 (0.679–1.340)
Early conversion	0.691 (0.462–1.035)	0.725 (0.480–1.096)	0.806 (0.532–1.223)
Late conversion	0.976 (0.592–1.611)	1.183 (0.707–1.982)	1.341 (0.798–2.254)
Late vs. early	1.413 (0.763–2.615)	1.632 (0.869–3.063)	1.653 (0.884–3.129)
*Rectum* ^^#^			
Postoperative complicated course			
OR	Ref	Ref	Ref
LR	**0.656 (0.594–0.724)**	**0.719 (647–0.799)**	**0.749 (0.671–0.837)**
CONV	**1.194 (1.009–1.413)**	1.080 (0.904–1.290)	1.118 (0.935–1.338)
Early conversion	1.064 (0.848–1.335)	0.977 (0.770–1.239)	1.015 (0.799–1.289)
Late conversion	**1.343 (1.073–1.679)**	1.196 (0.956–1.512)	1.233 (0.974–1.561)
Late vs. early	1.262 (0.936–1.7)	1.224 (0.898–1.669)	1.215 (0.891–1.657)
Mortality (in-hospital or < 30 days)			
OR	Ref	Ref	Ref
LR	0.482 (0.355–0.655)	**0.613 (0.440–0.855)**	0.784 (0.552–1.114)
CONV	0.914 (0.551–1.516)	0.880 (0.505–1.533)	1.084 (0.596–1.843)
Early conversion	0.647 (0.297–1.411)	0.546 (0.231–1.292)	0.666 (0.279–1.591)
Late conversion	1.203 (0.650–2.226)	1.305 (0.669–2.547)	1.504 (0.765–2.956)
Late vs. early	1.858 (0.724–4.765)	2.391 (0.855–6.682)	2.259 (0.803–6.352)

Bold values indicate statistically significant

*OR* open resection, *LR* laparoscopic resection, *CONV* laparoscopic conversion

*Added for the colon: location of tumor

**Year of operation

^^^The following factors were included in the multivariable model to correct for differences in casemix between patients; sex, age, ASA, Charlson Comorbidity Score, BMI, previous abdominal surgery, pre-operative tumor complications, pT-classification

^#^Added for the rectum: received radiotherapy (non, short course or chemoradiation), procedure (LAR, APR, or different), cT-classification, tumor distance from anal verge

For rectal cancer, no significant higher risk of a complicated course (adjusted odds ratio 1.118 CI 0.935–1.338) or mortality (adjusted odds ratio 1.084, CI 0.596–1.843) was found for the CONV group compared to OR.

An intra-operative complication compared to exposure difficulties as reason for conversion was significantly associated with a higher risk of a postoperative complicated course, with timing of conversion included in the multivariable model (adjusted odds ratio 2.282, CI 1.497–3.479) (Table S3).

With respect to pathological outcome, there were no significant differences between OR, LR, and CONV with respect to lymph node retrieval and R0 resection rates (Table 4). For rectal cancer, the CRM positivity rate was significantly lower after CONV compared to OR in univariate analysis (3 vs. 5%, respectively), with a similar rate compared to LR.

## Discussion

This population-based study showed an impressive increase in the use of a laparoscopic approach for resection of non-locally advanced, non-metastatic colorectal cancer in an elective setting to more than 80% in the Netherlands since 2010. Laparoscopic colorectal surgery in the Netherlands started with structured training and proctorship of a selected group of surgeons between 2003 and 2008 [24]. General colorectal surgeons learned essential laparoscopic skills during 24 elective laparoscopic colon resections under proctorship of an experienced laparoscopic surgeon. The trainee received a certificate after successfully completing the course, which was acknowledged by the Netherlands Health Care Inspectorate. Subsequently, these surgeons trained their colleagues and thereafter the residents. This study monitors the last steps of the implementation process. Conversion rates decreased below 10% for both colon and rectal cancer surgery, with a significant association between laparoscopic hospital volume and conversion rate. The distribution of early and late conversion did not change over time. For colon cancer, conversion was associated with a higher risk of a postoperative complicated course compared to a primary open approach, especially in case of late conversion due to intra-operative complications, be it without impact on mortality. With regard to complicated course and mortality, converted rectal cancer resections did not have a worse outcome compared to primary open resections.

Risk factors for conversion for different populations have been widely reported in the literature. Clancy et al. recently performed a meta-analysis of 15 studies and found an average conversion rate of 17.9% (± 10.1%) with male gender, rectal tumor, T3/T4 stage and node-positive disease as factors that negatively influence the completion of laparoscopic surgery [25]. After exclusion of locally advanced and metastatic disease as well as an emergency setting, conversion was also more often present in males in our study. For rectal cancer, this is presumably related to the narrower pelvis of men compared to women, while the reason for higher conversion rate in men with colon cancer is less clear. A possible explanation could be that men have more visceral fat [26], as Park et al. showed this risk factor to be associated with conversion [27]. Mid-rectal cancers had the highest risk of conversion, similar to the results of van der Pas et al. in the COLORII trial [28], and the explanation for this is still unclear. For colon cancer, the highest risk of conversion was the left hemicolectomy, what was also shown by Tekkis et al. [29] and Masoomi et al. [30], and is believed to be technically more challenging.

Conversion rates are expected to reduce over time. The CLASICC trial for example had a conversion rate of 34% [12] for rectal cancer, while this was 16% [28] in more recently published trials from Western populations. Surgical experience is one of the crucial elements for success in complex laparoscopic procedures. In the DSCA, specialization and volume of the individual surgeons are not registered, and therefore we used laparoscopic hospital volume to reflect the level of experience. A clear, casemix adjusted, association of laparoscopic hospital volume and the risk of conversion for both colon and rectum could be demonstrated. In literature, different cutoff points are used for laparoscopic volume and the risk of conversion. Husher et al. performed a prospective study on laparoscopic colorectal surgery outcomes in 10 high-volume centers, in which high volume was defined as > 100 colorectal resections including more than 40 laparoscopic resections [31]. Conversion rate was 10.5% with T4 patients being included in the study. In a systematic review, Miskovic et al. showed a plateau of the learning curve for conversion from 152 cases by using risk-adjusted CUSUM curves [32]. As the authors also mention, one could raise ethical questions with the protracted length of the learning curve. But in our view, early conversion is acceptable and could actually be considered as a good judgement of one’s own laparoscopic skills as long as it is not associated with intra-operative complications (i.e., reflecting a reactive conversion). For this reason, conversion is often not considered to be an appropriate quality measure. Massaroti et al. showed that, adjusted for patient and surgeon factors, training type (laparoscopic or open) was not associated with conversion rate [33]. The surgeons were classified in the high laparoscopic volume group when they had performed > 100 laparoscopic procedures. In our study, where data were aggregated at a hospital level, increased laparoscopic hospital volume also showed a significant decrease in conversion rate with all hospitals performing more than 300 procedures for colon cancer in the last 5 years having conversion rates around 10% (Fig. 1). For rectal cancer, conversion rates decrease to around 10% for hospitals who performed more than 150 laparoscopic rectal cancer resections during the study period.

For colon cancer, the odd on a laparoscopic approach was slightly higher in a teaching hospital compared to a non-teaching hospital. However, no differences in conversion rates were found after correcting for laparoscopic hospital volume, thereby confirming the laparoscopic volume–conversion relationship. However, in rectal cancer, also a slightly higher odd on laparoscopic approach was found in teaching hospitals compared to non-teaching hospitals, and this was accompanied by a slightly higher odd on conversion. In contrast, the odd on laparoscopic approach in rectal cancer was found to be lower in academic hospitals, with also a lower odd on conversion. One might hypothesize that expertise, different patient selection for a laparoscopic approach or a teaching environment are contributing factors to these observations. If a laparoscopic procedure is converted to open, Allaix et al. showed no significant differences in short-term postoperative morbidity, mortality, or hospital stay between the converted group compared to the laparoscopic completed group in a cohort of 1114 patients [34]. In contrast, even compared to the OR, we did find a significant higher short-term postoperative complicated course for the converted colon cancer resections, and late conversion (after 30 min) increased this risk. This did not translate into an increased risk of postoperative mortality. A recent meta-analysis did show a higher risk of 30-day mortality after conversion compared to completed laparoscopic resection [25]. However, we think that it is more appropriate to compare the converted group with primary open surgery. In a large national database of 207,311 colorectal resections for malignant as well as benign disease in the United States, conversion had a higher morbidity and mortality than completed laparoscopic procedures, but better outcome than primary open procedures [30]. The laparoscopic procedures were most likely performed by colorectal specialists, probably resulting in better outcomes after conversion than primary open procedures that were probably also performed by non-specialists. In the Netherlands, both elective open and laparoscopic procedures for cancer are nowadays performed by colorectal specialists, which might explain the similar outcome. In rectal cancer, we did not detect a significant impact on complicated course after conversion. A possible explanation for this observation could be that laparoscopic rectal cancer resections were often undertaken only after laparoscopic experience was already gained in colon cancer, thereby shortening the learning curve for rectal cancer.

Because conversion rates stabilize around 8%, it is important to know whether oncologic outcome is compromised in this subgroup. This could influence the choice of the surgical approach in patients with several risk factors for conversion. Unfortunately, the DSCA database does not include long-term oncological outcome, but the surrogate pathological outcome measures lymph node retrieval and radicality of the resection suggest no impact. Clancy et al. performed a systematic review with meta-analysis and found conversion of laparoscopic colorectal cancer resection to be associated with an increase in disease recurrence and overall mortality, but the patients in the converted groups more often had locally advanced disease [25]. Allaix et al. showed that converted patients had a worse 5-year overall survival (OS) and disease-free survival (DFS) in univariate analysis, in which patients with pathologic T4 tumors were included [34]. In multivariate analysis, however, only pathologic T4 stage and tumor-positive lymph node ratio > 0.25 were independently associated with OS and DFS. The prospective database study of Li et al. showed a similar 5-year DFS and OS in the converted group compared to the laparoscopic completed group and open resection group [35]. It is important to emphasize that pT4 stage was included in all three studies. T4 stage is more likely to be converted, especially if a multivisceral resection is needed [21]. For non-locally advanced disease, there seems not to be an oncological safety issue, but further studies are necessary to confirm this.

The strength of this study is the large numbers of patients and external validity related to the population-based data reflecting daily practice. But there are also some limitations. A certain degree of missing data is inevitable in population-based studies. Considering casemix adjustment, there is always a possibility that not all contributing factors were included. As mentioned earlier, the DSCA does not provide information on surgeon level. Also, we did not have any detailed information on the intent and type of the laparoscopic approach. For example, several hospitals started their experience with a short explorative laparoscopy without the intention to complete the procedure laparoscopically. Furthermore, recently robotic surgery has been introduced in the Netherlands, but this is not yet registered in the DSCA. As mentioned earlier, we were not able to use the exact definitions of the different types of conversion (strategic or reactive) as defined by Blikkendaal et al. [22], because these were not incorporated in the DSCA dataset during this study period. Finally, the DSCA does not provide information on disease-free survival and overall (long-term) survival, which is an important topic for conversion.

In conclusion, this population-based study showed that the laparoscopic approach has become standard of care for colorectal cancer resection in the Netherlands. With increasing laparoscopic hospital volume, conversion decreases below 10% with only minimal impact of conversion on short-term postoperative outcome. To perform an early conversion can be an appropriate decision, for which reason this type of conversion should not be considered a failure.

## Electronic supplementary material

Below is the link to the electronic supplementary material.

Supplementary material 1 (DOCX 37 KB)

Supplementary material 2 (DOCX 28 KB)

Supplementary material 3 (DOCX 36 KB)
