# Supplementary material for: Laparoscopic conversion in colorectal cancer surgery; is there any improvement over time at a population level?
Source: Surg Endosc. 2018 Jan 17;32(7):3234–46. doi: 10.1007/s00464-018-6042-2 (PMC5988765; doi:10.1007/s00464-018-6042-2)
Supplement: Supplementary file 1 — Supplementary material 1 (DOCX 37 KB) [file 464_2018_6042_MOESM1_ESM.docx]

|  |  | No. Of converted |  | **Odds ratio (CI)** | **Odds ratio (CI)** | |
| --- | --- | --- | --- | --- | --- | --- |
|  |  | Patients (%) |  | **Univariate** | **Multivariate** |  |
| **COLON** | **Surgical procedure** |  |  |  |  |  |
|  | Right hemicolectomy | 643 (9.8%) |  | Ref | Ref |  |
|  | Transversectomy | 30 (13%) |  | 1.412 (0.954-2.090) | 1.392 (0.922-2.102) | |
|  | Left hemicolectomy | 266 (16.5%) |  | **1.865 (1.598-2.188)** | **1.960 (1.670-2.300)** | |
|  | Sigmoidectomy | 658 (8.9%) |  | 0.914 (0.815-1.024) | 0.954 (0.846-1.076) | |
| **RECTUM^#** | **Distance from anal verge** |  |  |  |  |  |
|  | >10 cm | 187 (9.5%) |  | Ref | Ref |  |
|  | 6-10 cm | 407 (12.5%) |  | **1.369 (1.140-1.643)** | **1.329 (1.095-1.613)** | |
|  | <=5 cm | 220 (8.6%) |  | 0.896 (0.730-1.099) | 1.180 (0.918-1.516) | |
| ^The following factors were included in the multivariable model to correct for differences in case mix between patients; sex, age, ASA, charlson comorbidity score, BMI, previous abdominal surgery, pre operative complications, pT-classification, year of operation, **hospital volume and type of hospital** . #Added for the rectum: received radiotherapy (non, short course or chemoradiation) , procedure (LAR, APR or different), cT-classification. | | | | | | |
|  |  |  |  |  |  |  |
|  |  |  |  |  |  |  |
|  |  |  |  |  |  |  |

**Table S1: Uni- and multivariable analysis of the association of the surgical procedure (colon) and distance of tumor from anal verge (rectum) on conversion.**
