# Supplementary material for: Laparoscopic conversion in colorectal cancer surgery; is there any improvement over time at a population level?
Source: Surg Endosc. 2018 Jan 17;32(7):3234–46. doi: 10.1007/s00464-018-6042-2 (PMC5988765; doi:10.1007/s00464-018-6042-2)
Supplement: Supplementary file 2 — Supplementary material 2 (DOCX 28 KB) [file 464_2018_6042_MOESM2_ESM.docx]

|  |  | **Odds ratio (CI) Multivariate** | |
| --- | --- | --- | --- |
|  | **Reason of conversion** |  |  |
| **COLON^*** | Exposure difficulties | Ref |  |
|  | Extensiveness | 0.962 (0.633-1.461) | |
|  | Intra-operative complication | **2.282 (1.497 - 3.479)** | |
| **RECTUM^#** | Exposure difficulties | Ref |  |
|  | Extensiveness | 0.988 (0.562-1.736) | |
|  | Intra-operative complication | 1.711 (0.947-3.092) | |
| ^The following factors were included in the multivariable model to correct for differences in case mix between patients; sex, age, ASA, charlson comorbidity score, BMI, previous abdominal surgery, pre operative complications, pT-classification, **timing of conversion (early vs late), laparoscopic hospital volume and type of hospital**.**,** year of operation. *Added for the the colon: location of tumor. #Added for the rectum: received radiotherapy (non, short course or chemoradiation), procedure (LAR, APR or different), cT-classification, tumor distance from anal verge. | | | |
|  |  |  |  |
|  |  |  |  |
|  |  |  |  |
|  |  |  |  |

**Table S3.: Multivariate analysis for the association of the reason of conversion on complicated course.**
