# Supplementary material for: Laparoscopic conversion in colorectal cancer surgery; is there any improvement over time at a population level?
Source: Surg Endosc. 2018 Jan 17;32(7):3234–46. doi: 10.1007/s00464-018-6042-2 (PMC5988765; doi:10.1007/s00464-018-6042-2)
Supplement: Supplementary file 3 — Supplementary material 3 (DOCX 36 KB) [file 464_2018_6042_MOESM3_ESM.docx]

|  | Avarage Laparoscopic  hospital volume per year (2011-2015) | No. of Patients | No. of converted Patients (%) | **Odds ratio (CI)** | **Odds ratio (CI)** | |
| --- | --- | --- | --- | --- | --- | --- |
|  |  |  |  | **Univariate** | **Multivariate** |  |
| **COLON^*** | <30 | 4156 | 505 (12.1%) | Ref | Ref |  |
|  | 30-50 | 4098 | 450 (11.0%) | 0.893 (0.780-1.022) | 0.912 (0.776-1.071) | |
|  | >50 | 7959 | 688 (8.6%) | **0.685 (0.607-0.774)** | **0.718 (0.605-0.852)** | |
|  | >50 vs 30-50 |  |  | **0.768 (0.677-0.870)** | **0.788 (0.689-0.900)** | |
| **RECTUM^#** | <20 | 2745 | 372 (13.6%) | Ref | Ref |  |
|  | 20-30 | 2355 | 254 (10.8%) | **0.763 (0.644-0.904)** | **0.573 (0.465-0.707)** | |
|  | >30 | 2686 | 217 (8%) | **0.561 (0.470-0.669)** | **0.419 (0.338-0.520)** | |
|  | >30 vs 20-30 |  |  | **0.735 (0.608-0.889)** | **0.731 (0.374-0.814)** | |
| ^The following factors were included in the multivariable model to correct for differences in case mix between patients; sex, age, ASA, charlson comorbidity score, BMI, previous abdominal surgery, pre operative complications, pT-classification, year of operation  **and type of hospital**. *Added for the the colon: location of tumor. #Added for the rectum: received radiotherapy (non, short course or chemoradiation), procedure (LAR, APR or different), cT-classification, tumor distance from anal verge. | | | | | | |
|  |  |  |  |  |  |  |
|  |  |  |  |  |  |  |
|  |  |  |  |  |  |  |
|  |  |  |  |  |  |  |

**Table S2: Uni- and multivariable analysis of the association of hospital volume on conversion.**
